# HBV immunization and vaccine coverage among hospitalized children in Cameroon, Central African Republic and Senegal: a cross-sectional study

**DOI:** 10.1186/s12879-015-1000-2

**Published:** 2015-07-12

**Authors:** Claudine Bekondi, Roberta Zanchi, Abdoulaye Seck, Benoit Garin, Tamara Giles-Vernick, Jean Chrysotome Gody, Petulla Bata, Angèle Pondy, Suzie Moyo Tetang, Mamadou Ba, Chantal Same Ekobo, Dominique Rousset, Jean-Marie Sire, Sarah Maylin, Loïc Chartier, Richard Njouom, Muriel Vray

**Affiliations:** Laboratoire des Virus Oncogènes, Institut Pasteur de Bangui, rue Pasteur, BP 923 Bangui, République Centrafricaine; Unité d’Epidémiologie des Maladies Emergentes, Institut Pasteur, 25-28 rue du Docteur Roux, 75015 Paris, France; Laboratoire de Biologie Médicale, Institut Pasteur de Dakar, 36 Avenue Pasteur, Dakar, Sénégal; Institut Pasteur de Madagascar, BP 1274 Antananarivo, Madagascar; Services des soins intensifs, Complexe Pédiatrique, BP 911 Bangui, République Centrafricaine; Complexe Pédiatrique, BP 911 Bangui, République Centrafricaine; Centre Mère-Enfant, Fondation Chantal Biya, Yaoundé, Cameroun; Centre Hospitalier d’Essos, Yaoundé, Cameroun; Hôpital Albert Royer, Dakar, Sénégal; Laboratoire de Virologie, Institut Pasteur Guyane, 23 avenue Pasteur, BP 6010, 97306 Cayenne, France; Département de virologie, Hôpital Saint Louis, Paris, France; Institut National de la Santé et de la Recherche Médicale, Faculté de Médecine Paris-Diderot, Paris, France; Service de Microbiologie CHU St Louis, 75010 Paris, France; Service de Virologie, Centre Pasteur du Cameroun, P.O.Box 1274, Yaoundé, Cameroun

**Keywords:** HBV markers, HBV vaccine, African children, Cross-sectional survey

## Abstract

**Background:**

Hepatitis B is a major health concern in Africa. The vaccine against hepatitis B virus (HBV) was introduced into the Expanded Programme on Immunization (EPI) of Cameroon and Senegal in 2005, and of CAR (Central African Republic) in 2008. A cross-sectional study was conducted to assess HBV immunization coverage following the vaccine’s introduction into the EPI and factors associated with having been vaccinated.

**Methods:**

All hospitalized children, regardless of the reasons for their hospitalization, between 3 months and 6 years of age, for whom a blood test was scheduled during their stay and whose condition allowed for an additional 2 mL blood sample to be taken, and who provided the parent’s written consent were included. All children anti-HBs- and anti-HBc + were tested for HBsAg.

Vaccination coverage was assessed in three different ways: immunization card, maternal recall and serologic anti-HBs profile.

**Results:**

1783 children were enrolled between April 2009 and May 2010. An immunization card was only available for 24 % of the children. The median age was 21 months.

Overall HBV immunization coverage based on immunization cards was 99 %, 49 % and 100 % in Cameroon, CAR and Senegal, respectively (*p* < 0,001). The immunization rate based on maternal recall was 91 %, 17 % and 88 % in Cameroon, CAR and Senegal, respectively (*p* < 0,001). According to serology (anti-HBs titer ≥ 10 mUI/mL and anti-HBc-), the coverage rate was 68 %, 13 % and 46 % in Cameroon, CAR and Senegal, respectively (*p* < 0,001). In Senegal and Cameroon, factors associated with having been vaccinated were: mother’s higher education (OR = 2.2; 95 % CI [1.5–3.2]), no malnutrition (OR = 1.6; 95 % CI [1.1–2.2]), access to flushing toilets (OR = 1.6; 95 % CI [1.1–2.3]), and < 24 months old (OR = 2.1; 95 % CI [1.3–3.4] between 12 and 23 months and OR = 2.7; 95 % CI [1.6–4.4] < 12 months). The prevalence of HBV-infected children (HBsAg+) were 0.7 %, 5.1 %, and 0.2 % in Cameroon, CAR and Senegal, respectively (*p* < 0.001).

**Conclusions:**

Assessing immunization coverage based on immunization cards, maternal recall or administrative data could be usefully reinforced by epidemiological data combined with immunological profiles. Serology-based studies should be implemented regularly in African countries, as recommended by the WHO. Malnutrition, lack of maternal education and poverty are factors associated with vaccine non-compliance. The countries’ vaccination programs should actively address these problems.

**Electronic supplementary material:**

The online version of this article (doi:10.1186/s12879-015-1000-2) contains supplementary material, which is available to authorized users.

## Background

Infection with hepatitis B virus (HBV) is a public health problem worldwide [1], with more than 350 million chronic carriers [2], 25–30 % of whom will die from the consequences of chronic infection [1].

In sub-Saharan Africa, contact with HBV, as measured by the prevalence of anti-HBc antibodies, varies from 65 to 85 % [3]; HBsAg prevalence ranges from 9 to 20 % [4], and the predominant source of HBV transmission is horizontal [5].

Antiviral treatment can reduce morbidity and mortality, but access remains limited in developing countries. Prevention of HBV by vaccination is a key and often the sole control strategy [6].

The anti-HBV vaccine is well tolerated and highly immunogenic in all age groups [7]. In 1991, the Global Advisory Group of the EPI (Expanded programme on immunization) recommended integrating it into all national immunization programmes [8]. For more than a decade, the Global Alliance for Vaccines and Immunization (GAVI) has actively supported HBV vaccination in eligible countries throughout the world.

Cameroon, Central African Republic (CAR) and Senegal present high HBV endemicity. In Cameroon, the prevalence of HBsAg positivity varies from 7.7 % among pregnant women [9, 10] to 25 % in children > 4 years of age [11]. CAR reports 14 % HBsAg-positivity among young adults [12], 15 % among hospitalized patients in Bangui [13] and 10.6 % in rural areas [14]. In Senegal, the HBV chronic infection prevalence varies between 7 % among newborns [15] to 17 % among blood donors [16].

In response to WHO recommendations, Cameroon and Senegal introduced the vaccine into their EPIs in 2005 and CAR in 2008. All three countries administer three doses of the pentavalent vaccine (Zilbrix™, a DTPw-HBV combination vaccine [17], in Cameroon and CAR, QuinvaxemTM, a DTwP-HepB-Hib vaccine, in Senegal) to infants at 6, 10 and 14 weeks of age. At the time of our study, the monovalent HBV vaccine was not available in the three countries, and thus infants did not receive a birth dose, even though universal vaccination with Hepatitis B at birth is a current recommendation [1].

We conducted a cross-sectional study in a selected population of hospitalized children in the sub-Saharan African capitals of Cameroon, CAR and Senegal. On the basis of this study, we previously published an article highlighting the low protection rate (58 %) in Senegal compared to Cameroon (92 %) among children immunized with three injections of anti-HBV vaccine and in possession of an immunization card [18].

Here we reported results on i) HBV immunization coverage using different methods and HBV infection in a selected population of hospitalized children in the capitals of Cameroon, CAR and Senegal, ii) factors associated with having been vaccinated in Senegal and Cameroon in this selected population.

## Methods

### Study population

A cross-sectional study was conducted in five children’s hospitals: one hospital in Bangui (CAR), two in Yaoundé (Cameroon) and two in Dakar (Senegal). Both the Yaoundé and Dakar study sites included one pediatric hospital treating infants from families of impoverished socioeconomic status, and one general hospital serving a relatively well-off population. The Bangui site was a pediatric hospital serving children from families of all socioeconomic levels living in the city and its outskirts.

Between April 2009 and May 2010, children aged 3 month to 6 years, hospitalized for any reason, with a blood sample prescribed during hospitalization, health conditions allowing an extended blood sample between 2 mL and 5 mL according to the age. Children were consecutively enrolled after their parents or legal guardians received an information notice and oral explanation in the local language and provided a written consent.

### Ethical approval

This study was approved by the Senegal Health Research National Council, the National Ethics Committee of Cameroon and the Scientific Committee responsible for validation protocols and study results in Central African Republic.

### Data collection

Data collected were: i) general characteristics (age, sex, weight), ii) clinical features (reasons for hospitalization, vaccination records on the immunization card), iii) socio-economic characteristics (place of residence, number of people in the household, mother’s education (higher level: at least primary education), personal transportation, electricity, running water, toilets type) and iv) serological data (anti-HBs antibodies, anti-HBc antibodies, HBsAg, HBeAg) and HBV DNA, when the child was HBsAg-positive.

If the enrolled child’s immunization card was available, vaccination against HBV and dates of vaccination were recorded. Otherwise, the mother was asked about the child’s vaccination status.

Complete vaccination was defined as having received all three injections according to the vaccination card in compliance with the WHO vaccination schedule (6, 10 and 14 weeks of age). Partial vaccination was defined as having received one or two doses according to the immunization card, regardless of the immunization schedule.

Nutritional status was estimated separately for boys and girls by the Z-score, calculated on the weight for age, according to WHO standards for children between 3 and 60 months old, and to CDC standards for older children. Moderate or severe malnutrition was defined as a Z-score ≤ −2 SD [19–21].

### HBV markers

All samples were tested for anti-HBc and quantified for anti-HBs by Enzyme ImmunoAssay (EIA) (DiaSorin Biomedica, Sallugia, Italy). The correlate of protection for HBV is an anti-HBs titer ≥10 mIU/mL [22, 23]. All children anti-HBs-negative and anti-HBc-positive were tested for HBsAg by automated EIA (AxSYM, Abbott laboratories, Chicago, USA). All HBsAg-positive children’s mothers were called by phone so that children could be retested free of charge six months later. Viral loads were measured by the Cobas AmpliPrep/Cobas TaqMan HBV assay, v2.0 (Roche Diagnostics, Meylan, France) at Saint-Louis Hospital. The limit of detection was 20 IU /mL. Except for viral load quantification, all laboratory tests were performed in each country.

### Statistical analysis

The children’s characteristics were described as medians and interquartile ranges (IQR) for continuous variables and percentages for discrete variables.

For univariate and multivariate analysis, quantitative variables were expressed as dichotomous variables using either the median or a clinically relevant threshold.

Univariate analysis was based on the Fisher’s exact test for discrete variables and by analysis of variance or the Kruskal-Wallis test for continuous variables. All variables associated with “having been vaccinated” in univariate analysis (*p* < 0.25) were included in a backward stepwise logistic regression model. A p value of ≤0.05 was considered statistically significant. Adequacy of the model was established through the Hosmer Lemeshow tests. Interactions between the variables found to be associated with “having been vaccinated” in the univariate analysis were tested using likelihood-ratio test. Our data on immunization coverage estimated by serological markers in children born one year after integration of vaccine into EPI (2005 in Cameroon and Senegal, and 2008 in CAR) were compared with data reported by WHO on immunization coverage of surviving infants between 2006 and 2009 [24–26].

Data were analyzed using STATA software version 12.0 (Stata Corporation, College Station, Texas).

### Selected populations for analysis

Using the only publication showing that it is possible to distinguish between the passive transfer of maternal anti-HBc and HBV exposure in children ≥12 months [27], we divided subjects between children younger than 12 months and those older than 12 months.

1. To evaluate the anti-HBV vaccination coverage from serology analysis, anti-HBs + and anti-HBc + children < 12 months were removed: their vaccination status could not be determined, since anti-HBs antibodies can be derived from the mother. Anti-HBs + and anti-HBc- children, regardless of age, were considered vaccinated and protected for an anti-HBs level titer ≥10 mIU/mL, assuming that most sub-Saharan African mothers were unvaccinated [10].
2. To evaluate vaccination coverage by combining serology and vaccination card documentation: those considered to be vaccinated were children with serological protection (anti-HBs titer ≥10 mIU/mL), as well as children who were unprotected based on serology, but who had received a complete HBV vaccination according to their immunization cards.
3. To evaluate factors associated with having been vaccinated against HBV (regardless of serological status, protection or non-protection): an analysis was conducted in Cameroon and Senegal for children born in 2006 and after, i.e., at least one year after the integration of the HBV vaccine into the EPIs. CAR children were not included because the vaccine was integrated later. The variable “vaccinated” implied having received a complete HBV vaccination according to the immunization card, or if no immunization card existed, by anti-HBs + and anti-HBc- status (with anti-HBs titer ≥10mIU/mL). All anti-HBs + and anti-HBc + children with no immunization card were removed because we could not know whether they were vaccinated but not protected, or if they were not vaccinated at all.
4. HBV current infection was estimated by the number of children with HBsAg-positivity.

## Results

### General characteristics of the children

**Table 1:** Characteristics of children and their families

	Cameroon n = 763	CAR n = 535	Senegal n = 485	Total n = 1783	*p*
Sex, female	329 (43)	247 (46)	212 (44)	788 (44)	0.54
Age (months) ^a^ ^13^	17 [10; 33]	27 [19; 37]	20 [10; 36]	21 [12; 36]	<0.001
Immunization card (availability) ^123^	177 (23)	41 (8)	210 (43)	428 (24)	<0.001
Complete^b^	166 (22)	13 (2)	172 (35)	351 (20)	<0.001
Partial^c^	10 (1)	7 (1)	38 (8)	55 (3)	
Moderate or severe malnutrition^23^ (yes)	104 (14)	68 (13)	225 (46)	397 (23)	<0.001
Reasons for hospitalisation					<0.001
Gastro-intestinal infection	172 (23)	103 (19)	108 (22)	383 (21)	
Respiratory infection	117 (15)	42 (8)	137 (28)	296 (17)	
Malaria	91 (12)	289 (54)	12 (2)	392 (22)	
Other infectious syndrome	253 (33)	39 (7)	59 (12)	351 (20)	
Other	130 (17)	62 (12)	169 (35)	361 (20)	
Place of residence ^123^ (capital)	723 (95)	441 (82)	127 (26)	1291 (72)	<0.001
No of people per household^a^ ^123^	6 [4; 8]	8 [5; 10]	12 [7; 18]	7 [5; 10]	<0.001
Household ≥ 7 people ^123^	287 (38)	329 (62)	367 (76)	983 (55)	<0.001
Mother’s education ^123^ (higher level^d^)	751 (99)	489 (92)	242 (51)	1482 (84)	<0.001
Personal transportation ^13^ (Car or motorcycle)	149 (20)	48 (9)	70 (14)	267 (15)	<0.001
Electricity (yes) ^123^	754 (99)	227 (42)	424 (87)	1405 (79)	<0.001
Running water (yes) ^123^	382 (50)	62 (12)	411 (85)	855 (48)	<0.001
Flushing toilets inside house ^123^ (yes)	327 (43)	18 (3)	86 (18)	431 (24)	<0.001

^a^ Median [IQ1; IQ3]

^b^ three injections in the immunization schedule

^c^ at least one injection

^d^ at least primary education

1: significant difference between Cameroon and CAR (*p* ≤ 0.05)

2: significant difference between Cameroon and Senegal (*p* ≤ 0.05)

3: significant difference between Senegal and CAR (*p* ≤ 0.05)

A total of 1783 children were recruited: 763 in Cameroon, 535 in CAR and 485 in Senegal. No mothers of eligible children refused to participate in the study.

General characteristics of the children are summarized in Table 1. Forty-four percent of the children were female, and the median age was 21 months [12–36 months]; children were significantly older in CAR.

The main causes of the children’s hospitalization were other infectious syndrome (33 %) and gastro-intestinal infections (23 %) in Cameroon; malaria in CAR (54 %); and respiratory infections (28 %) and other reasons (35 %) in Senegal.

Immunization cards were available for 24 % of all children. Senegalese subjects possessed a higher percentage of immunization cards (43 %) compared to those in Cameroon (23 %) and CAR (8 %), (*p* < 0.001). Among the 1355 children without an available immunization card, maternal recall of HBV immunization was documented for 636 children (79, 460 and 97 children in Cameroon, CAR and Senegal, respectively).

In Senegal, 51 % of the mothers had received primary or secondary education, compared to 99 % in Cameroon and 92 % in CAR (*p* < 0.001). Senegalese children suffered significantly more from malnutrition (46 %) than in Cameroon (14 %) and CAR (13 %), (*p* < 0.001). In CAR, families lived under poorer conditions than elsewhere.

Comparisons between the two Cameroonian hospitals and between the two Senegalese hospitals confirm the different socioeconomic levels of the populations served. For instance, at one Cameroonian hospital, 65 % (173/265) of the families had indoor flushing toilets, whereas only 31 % (154/496) of those at the other hospital had them. Similarly, in Senegal, 50 % (21/42) of patients attending one hospital came from families with indoor flushing toilets, compared with just 15 % (65/443) at the other. Concerning access to running water, our results resembled those pertaining to indoor flushing toilets: the two Cameroonian hospitals serving 72 % (190/265) compared to 39 % (192/497) of families whose homes had piped water. Overall, our subjects came from a wide range of families, from well-off functionary families to very poor ones.

### HBV serological markers 

**Table 2:** HBV serological markers N (%)

		Cameroon n = 763	CAR n = 535	Senegal n = 485	Total n = 1783	*p*
Anti-HBc- (N = 1437)	Anti-HBs ≥ 10 mIU/mL	482 (63)	67 (13)	206 (42)	755 (42)	<0.001
	Anti-HBs < 10 mIU/mL	176 (23)	331 (62)	175 (36)	682 (38)	<0.001
Anti-HBc + (N = 346)	Anti-HBs ≥ 10 mIU/mL	80 (10)	35 (7)	65 (13)	180 (10)	0.001
	Anti-HBs < 10 mIU/mL	25 (3)	102 (19)	39 (8)	166 (10)	<0.001
	- HBsAg-positive	5	27	1	33	
	- HbsAg-negative	20	75	38	133	

HBV serological markers are summarized in Table 2. Forty-two percent (755/1783) of children were anti-HBs titer ≥10 mIU/mL and anti-HBc-: Cameroon had a significantly higher percentage than did CAR and Senegal (63 %, 13 % and 42 %, respectively, *p* < 0.001).

Thirty-eight percent (682/1783) of children were anti-HBs titer <10 mIU/mL and anti-HBc-. Cameroon and Senegal had a significantly lower proportion of children anti-HBs titer <10 mIU/ml and anti-HBc- (23 % and 36 %, respectively), compared to the 62 % of children observed in CAR, *p* < 0.001).

Among the 346 children anti-HBc+, 33 were HBsAg-positive. The remaining 313 children were anti-HBs titer ≥10 mIU/mL and anti-HBc + (*n* = 180), or anti-HBs titer <10 mIU/mL and anti-HBc+, HBsAg-negative (*n* = 133). Sixty percent (188/313) of these children were ≥12 months.

### Vaccination coverage according to immunization card, maternal recall and serology 

**Table 3:** HBV vaccination coverage according to immunization card, maternal recall or serology: n/N (%)

	Cameroon n = 763	CAR n = 535	Senegal n = 485	Total n = 1783	*p*
Immunization card (availability) ^123^	177 (23)	41 (8)	210 (43)	428 (24)	<0.001
Vaccinated^123a^	176/177 (99)	20/41 (49)	210/210 (100)	406/428 (95)	<0.001
Complete vaccination^b^	166	13	172	351	
Partial vaccination^c^	10	7	38	55	
Anti-HBs titer ≥10 mIU/mL	156/176 (89)	17/20 (85)	128/210 (61)	301/406 (74)	
Maternal recall ^123 d^	72/79 (91)	80/460 (17)	85/97 (88)	237/636 (37)	<0.001
Anti-HBs titer ≥10 mIU/mL	54/72 (75)	47/80 (59)	65/85 (76)	166/237 (70)	
Serology ^123^ (Anti-HBs titer ≥10 mIU/mL^12^ and Anti-HBc-) ^e^	482/712 (68)	67/534 (13)	206/444 (46)	755/1690 (45)	<0.001
Immunization card (complete vaccination) or serology ^f^	527/737 (72)	68/535 (13)	294/467 (63)	889/1739 (51)	<0.001

^a^ received at least one dose injection

^b^ received all three doses injections

^c^ received one or two doses injections

^d^ documented for 636 children without available immunization card

^e^ children < 12 months excluded (n = 93)

^f^ children < 12 months without available immunization card excluded (n = 44)

1: significant difference between Cameroon and CAR (*p* ≤ 0.05)

2: significant difference between Cameroon and Senegal (*p* ≤ 0.05)

3: significant difference between Senegal and CAR (*p* ≤ 0.05)

HBV vaccination coverage according to immunization card, maternal recall or serology are shown in Table 3. Among these 428 children who had an immunization card, 9 were vaccinated outside of the official vaccination programme (EPI) (7 with complete vaccination and 2 with partial vaccination). Almost all Cameroonian and Senegalese children had been vaccinated (with partial or complete vaccination) (99 % and 100 %, respectively), exceeding that of CAR (49 %). Children with an immunization card tended to be younger, 13 months versus 21 months for the entire study population, (*p* = 0.006).

The delay between vaccination doses revealed adherence to the recommended schedule: the median time between birth and the first vaccine dose was 47 days (28- 66); between the first and second dose, 32 days (28- 29); and between the second and third dose, 32 days (29, 30). Among the children vaccinated according to the immunization card, Cameroon displayed the highest percentage of children with complete vaccination (94 % (166/176) against 65 % (13/20) in CAR and 82 % (172/210) in Senegal. Seventy-four percent (301/406) had an anti-HBs titer ≥10 mIU/mL (89 %, 85 % and 61 % in Cameroon, CAR and Senegal, respectively).

On the basis of the 636 maternal recalls, estimates of immunization coverage reached 37 % overall. In Cameroon and Senegal, coverage (91 % and 88 %, respectively) was substantially higher than in CAR, *p* < 0.001.

Seventy percent (166/237) of children reported as vaccinated by their mothers were protected based on serological data (anti-HBs titer ≥10 mIU/mL): 75 % in Cameroon, 59 % in CAR and 76 % in Senegal.

To estimate the immunization coverage from serological results, 93 anti-HBs + and anti-HBc + children < 12 months were removed: their vaccination status could not be determined since anti-HBs antibodies can be derived from the mother. On the basis of only serological results (anti-HBs titer ≥10 mIU/mL and anti-HBc-), overall immunization coverage was 45 % (755/1690). In Cameroon, coverage (68 %) was significantly higher than in CAR (13 %) and Senegal (46 %), (*p* <0.001). When immunization coverage was estimated both for serological results as defined above and, for children who were unprotected based on serology, from complete HBV vaccination according to immunization cards, vaccination coverage reached 51 % (889/1739) (44 children were excluded because they were anti-HBs + and anti-HBc + < 12 months without immunization card).

While the two approaches led to similar results in Cameroon and CAR, a difference of 17 % (46 % versus 63 %) was observed between the two estimates in Senegal.

### HBV immune protection (anti-HBs titer ≥10 mIU/mL among anti-HBc-) according to year of birth and country and comparisons with WHO estimates 

**Fig. 1:**
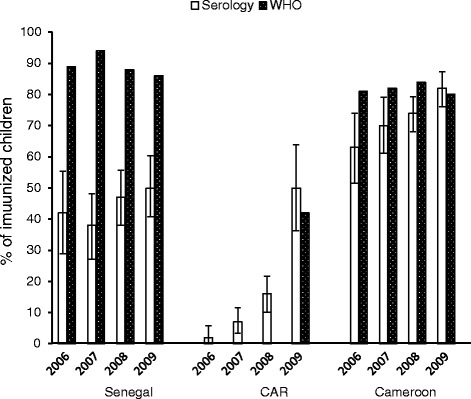
HBV immune protection (anti-HBs + ≥10 mIU/ml among anti-HBc-) according to the year of birth and the country compared to WHO coverage estimates. Y axis: percentage of immunized children

The percentages of children serologically protected gradually increased between 2006 and 2009 from 63 % (47/75) to 82 % (151/185) in Cameroon and from 2 % (2/83) to 50 % (27/54) in CAR. In Senegal, however, the percentage of children serologically protected fluctuated between 42 % (24/57) and 50 % (52/103) during these same years (Fig. 1).

### Factors associated with the fact of having been vaccinated

**Table 4:** Factors associated with having been vaccinated for children born in 2006 or later in Cameroon or Senegal

N (%)	Non-vaccinated^a^	Vaccinated^b^	Univariate analysis		Multivariate analysis	
	(n = 244)	(n = 731)	OR [CI_95%_]	*P*	OR [CI_95%_]	*p*
Mother’s education						
No education	79 (33)	101 (14)	1		1	
Higher education (at least elementary)	161 (67)	625 (86)	3.0 [2.2;4.3]	<0.001	2.2 [1.5;3.2]	<0.001
Nutritional status						
Malnutrition	93 (39)	169 (24)	1		1	
No malnutrition	147 (61)	539 (76)	2.0 [1.5;2.8]	<0.001	1.6 [1.1;2.2]	0.011
Age						
<12 months	67 (27)	269 (37)	2.5 [1.5; 3.9]	<0.001	2.7 [1.6;4.4]	<0.001
[12–24]	81 (33)	271 (37)	2.0 [1.3; 3.2]	0.002	2.1 [1.3;3.4]	0.003
[24–36]	55 (23)	124 (17)	1.4 [0.8; 2.3]	0.21	1.4 [0.8;2.4]	0.21
≥36 months	41 (17)	67 (9)	1		1	
Toilets						
Without flushing	190 (78)	453 (62))	1		1	
With flushing	54 (22)	277 (38)	2.2 [1.5;3.0]	<0.001	1.6 [1.1;2.3]	0.009

^a^ Children anti-HBs- and anti-HBc- or anti-HBs- and anti-HBc+

^b^ Children anti-HBs + and anti-HBc-, or based on the immunization card, with complete vaccination

In Cameroon and Senegal, four factors were identified as independently associated with having been vaccinated: mother’s higher level of education (OR = 2.2), no malnutrition (OR = 1.6), access to flushing toilets (OR = 1.6), and being <24 months old (OR = 2.1 between 12 and 23 months and OR = 2.7 < 12 months) (Table 4). There was no evidence for the interactions between the variables associated with having been vaccinated.

### HBV infections

The percentage of HBV-infected children was significantly lower in Cameroon [0.7 % (5/763)] and Senegal [0.2 % (1/485)] compared to CAR [5.1 % (27/535)] (*p* < 0.001). The overall median viral load was 8 log IU/mL. Among the 27 HBsAg-positive children in CAR, 14 (52 %) were IgM anti-HBc + and 20 (74 %) were HBeAg-positive. Among the twelve children who returned six months later, six were HBsAg-positive. In Cameroon and Senegal, among the six HBsAg-positive children, the two children who returned six months later were confirmed to be HBsAg-positive. In CAR, 20 out of 27 infected children were ≥24 months, indicating that they were infected before the HBV vaccine’s introduction into the EPI. Among the seven children <24 months, only one 15 months old child possessed an immunization card documenting that he was not vaccinated. No information about the vaccination status of six other children was available, but all were born before September 2008. In Cameroon, among the five children HBsAg-positive, only one was under 24 months old; no information about the child’s vaccine status was available. The other four children were born prior to July 2005. The only Senegalese child infected was born at the end of 2006.

## Discussion

Immunization coverage was estimated using different methods: the immunization card, maternal recall and serological profile, and a combination of immunization card and serology. Immunization rates calculated from serology showed higher coverage in Cameroon (68 %) and Senegal (46 %) than in CAR (13 %), because of the very recent introduction of the HBV vaccine in CAR. In Cameroon and CAR, the percentage of children immunized and protected increased over time from 2006 to 2009. This increased coverage relates to the level of investment and time required to set up the necessary delivery infrastructures and to reach isolated areas [31]. In both countries, results based on both vaccination and anti HBs profile convene closely to those based exclusively on serology, confirming that children vaccinated according to the immunization cards are protected.

In Senegal, the percentage of children vaccinated and protected fluctuated over time, with a maximum of 50 % in 2009 and a minimum of 38 % in 2007. If the results of subjects’ immunization cards are factored in, some children vaccinated according to the immunization cards were not, in fact, protected. Possible hypotheses for this lack of response to the vaccine, including storage conditions, cold chain issues or immunogenicity of the different vaccines, were previously reported [18].

The WHO/UNICEF, in assessing national immunization coverage of surviving infants, draws from several information sources: administrative data (reports from vaccination services), official data (the best estimates from authorities, which account for administrative data and any other available information) and monitoring survey data [29]. As shown in Fig. 1, in Cameroon, immunization coverage rates reported by the WHO for 2006–2009 (81 % to 88 %) are close to our results [23]. Similarly, in CAR, the WHO estimated coverage at 42 % in 2009, a rate similar to our results [24]. In Senegal, the WHO, relying on administrative and official data, estimated coverage at 89 % in 2006, 94 % in 2007, and 88 % in 2008 [25]. Serological analysis in our study produced substantially lower results. An external review of the EPI and a MICS (Multiple Indicator Cluster Survey) was carried out in Senegal in 2009 and reported immunization coverage of 74 % and 83 %, but the WHO did not use the results in its final estimates. Our results differed from WHO estimates when coverage analysis was based on serology results (50 %). These disparities may have different explanations. The WHO has noted that administrative data are subject to biases that can distort immunization coverage calculations [29], for example, when they include all vaccines delivered to vaccination centres, and not just vaccinations among children adhering to the immunization schedule [32]. Although vaccination is free, non-adherence to vaccination occurs in Senegal and Cameroon. We identified four factors associated with having been vaccinated against HBV (regardless of protection): mother’s higher education, having access to indoor flushing toilets, a child not being malnourished and being younger. The gradual increase in coverage following the vaccine’s introduction explains the association with age. Some studies have shown a link between mother’s education and acceptance of immunization [33]. Regarding the association between non-vaccination and malnutrition, studies have established a link between poor health and delayed vaccination of children [34–36]. The association with lack of indoor flushing toilets can be explained by families’ lower socio-economic status, entailing, for example, distance to a vaccination centre and transport costs [30].

The low rate of HBsAg carriers (0.7 % in Cameroon and 0.2 % in Senegal) suggests the effectiveness of the countries’ EPIs and of GAVI’s support for HBV vaccination in these two countries. Nevertheless, a significant number of children who are 12 months or more (220/1348); 9 % (46/527) in Cameroon and 13 % (45/341) in Senegal came into contact with the virus (anti-HBc+), suggesting the importance of improving hepatitis B vaccine coverage. Moreover, as recommended by the WHO, advancing the first dose within 24 h of birth could further reduce contact with the virus in these populations.

Our study has limitations. This hospitalized-based population from whom a blood sample was collected does not constitute a representative sample and cannot be considered representative of the entire child population. Unlike studies using nationally representative data from the household-based Demographic and Health Surveys (DHS) or two-stage cluster sampling methodology, we have studied only children hospitalized in five hospitals in three capitals. The selected hospitals, which serve different socioeconomic populations, probably do not significantly over- or underestimate immunization coverage and the generalizability of the serological findings. But it remains difficult to determine how much these findings differ from immunization coverage among rural populations. Canavan et al. recently show that in East Africa, urban residence is associated with increased odds of complete vaccination status [28].

The selection of hospitalized children, including 23 % of moderate or severe malnourished children may also have affected these estimates. In addition, the cross-sectional design allows us to draw conclusions about associations with complete vaccination, but causality cannot be ascertained from our data. Serology estimates did not account for children with anti-HBs + and anti-HBc + under 12 months old (93/1783). However, 53 % (49/93) of these children had an immunization card documenting them as vaccinated. We argue that the exclusion of 44 children had a limited impact on our overall results. In contrast, the strength of our study is that our Pasteur team evaluated the serological markers of samples from children admitted to the five capital city hospitals.

## Conclusion

Five years after the integration of the hepatitis B vaccine in their EPI, vaccination programmes must persist in improving vaccination coverage. Assessing immunization coverage based on immunization cards, maternal recall or administrative data could be usefully reinforced by epidemiological data combined with immunological profiles. Serology-based studies should be implemented regularly in African countries, as recommended by the WHO. Among the African populations studied, malnutrition, lack of maternal education and poverty are factors associated with vaccine non-compliance. The countries’ vaccination programs should actively address these problems.

### Ethical approval

The study was conducted in conformity with country regulations concerning ethical review and informed consent.

## Additional file

Additional file 1:
**STROBE Statement—checklist of items that should be included in reports of observational studies.**
