# Supplementary material for: HBV immunization and vaccine coverage among hospitalized children in Cameroon, Central African Republic and Senegal: a cross-sectional study
Source: BMC Infect Dis. 2015 Jul 12;15:267. doi: 10.1186/s12879-015-1000-2 (PMC4499446; doi:10.1186/s12879-015-1000-2)
Supplement: Additional file 1: — STROBE Statement—checklist of items that should be included in reports of observational studies. [file 12879_2015_1000_MOESM1_ESM.docx]

STROBE Statement—checklist of items that should be included in reports of observational studies

|  | Item No. | Recommendation | Page  No. | Relevant text from manuscript |
| --- | --- | --- | --- | --- |
| **Title and abstract** | 1 | (*a*) Indicate the study’s design with a commonly used term in the title or the abstract | Title | A cross sectional study |
|  |  | (*b*) Provide in the abstract an informative and balanced summary of what was done and what was found |  | Vaccination coverage was assessed in 3 different ways: immunization card, maternal recall and serologic anti-HBs profile in 1783 children hospitalized in Cameroon, Senegal and CRA. According to serology the coverage rate was 68%, 13% and 46% in Cameroon, CAR and Senegal, respectively (p<0,001).In Senegal and Cameroon, factors associated with having been vaccinated were: mother's higher education no malnutrition, access to flushing toilets and age. HBV-infected children were 0.7%, 5.1%, and 0.2% in Cameroon, CAR and Senegal, respectively (p<0.001). |
| Introduction | | | |  |
| Background/rationale | 2 | Explain the scientific background and rationale for the investigation being reported | Page 4 |  |
| Objectives | 3 | State specific objectives, including any prespecified hypotheses | Page 5 | HBV immunization coverage using different methods and HBV infection in a selected population of hospitalized children in the sub-Saharan African capitals of Cameroon, CAR and Senegal  Factors associated with having been vaccinated in Senegal and Cameroon in this selected population. |
| Methods | | | |  |
| Study design | 4 | Present key elements of study design early in the paper | Page 5 | Cross-sectional study in 5 children’s hospitals |
| Setting | 5 | Describe the setting, locations, and relevant dates, including periods of recruitment, exposure, follow-up, and data collection |  | Between April 2009 and May 2010, all children hospitalized in one of the five children's hospitals: one hospital in Bangui (CAR), two in Yaoundé (Cameroon) and two in Dakar (Senegal). |
| Participants | 6 | (*a*) *Cohort study*—Give the eligibility criteria, and the sources and methods of selection of participants. Describe methods of follow-up  *Case-control study*—Give the eligibility criteria, and the sources and methods of case ascertainment and control selection. Give the rationale for the choice of cases and controls  *Cross-sectional study*—Give the eligibility criteria, and the sources and methods of selection of participants | Page 5 | Consecutive children aged three month to six years, hospitalized for any reason, with a blood sample prescribed during hospitalization, health conditions allowing an extended blood sample between 2 mL and 5 mL according to the age. Children were consecutively enrolled after the parents or legal guardians received an information notice and oral explanation in the local language and provided a written consent |
|  |  | (*b*) *Cohort study*—For matched studies, give matching criteria and number of exposed and unexposed  *Case-control study*—For matched studies, give matching criteria and the number of controls per case |  |  |
| Variables | 7 | Clearly define all outcomes, exposures, predictors, potential confounders, and effect modifiers. Give diagnostic criteria, if applicable | Pages 5-8 | -general characteristics (age, sex, weight)  - clinical features (reasons for hospitalization, vaccination records on the immunization card)  -socio-economic characteristics (place of residence, number of people in the household, mother’s education (higher level: at least primary education), personal transportation, electricity, running water, toilets type)  -serological data (anti-HBs antibodies, anti-HBc antibodies, HBsAg, HBeAg) and HBV DNA, when the child was HBsAg+.  If the enrolled child’s immunization card was available, vaccination against HBV and dates of vaccination were recorded. Otherwise, the mother was asked about the child’s vaccination status.  Complete vaccination was defined as having received all three injections according to the vaccination card in compliance with the WHO vaccination schedule (6, 10 and 14 weeks of age). Partial vaccination was defined as having received one or two doses according to the immunization card, regardless of the immunization schedule. |
| Data sources/ measurement | 8* | For each variable of interest, give sources of data and details of methods of assessment (measurement). Describe comparability of assessment methods if there is more than one group | Pages 5-8 | If the enrolled child’s immunization card was available, vaccination against HBV and dates of vaccination were recorded. Otherwise, the mother was asked about the child’s vaccination status.  Complete vaccination was defined as having received all three injections according to the vaccination card in compliance with the WHO vaccination schedule (6, 10 and 14 weeks of age). Partial vaccination was defined as having received one or two doses according to the immunization card, regardless of the immunization schedule.  Nutritional status was estimated separately for boys and girls by the Z-score, calculated on the weight for age, according to WHO standards for children between 3 to 60 months old, and to CDC standards for older children. Moderate or severe malnutrition was defined as a Z-score ≤-2 SD [18-20]. |
| Bias | 9 | Describe any efforts to address potential sources of bias |  | NA |
| Study size | 10 | Explain how the study size was arrived at |  | NA |

Continued on next page

| Quantitative variables | 11 | Explain how quantitative variables were handled in the analyses. If applicable, describe which groupings were chosen and why | Page 8 | **Selected populations for analysis**  Using the only publication showing that it is possible to distinguish between the passive transfer of maternal anti-HBc and HBV exposure in children ≥12 months [26], we divided subjects between children younger than 12 months and those older than 12 months.  1) To evaluate the anti-HBV vaccination coverage from serology analysis, anti-HBs+ and anti-HBc+ children < 12 months were removed: their vaccination status could not be determined, since anti-HBs antibodies can be derived from the mother. Anti-HBs+ and anti-HBc- children, regardless of age, were considered vaccinated and protected for an anti-HBs level titer ≥10 mIU/mL, assuming that most sub-Saharan African mothers were unvaccinated [10].  2) To evaluate vaccination coverage by combining serology and vaccination card documentation: those considered to be vaccinated were children with serological protection (anti-HBs titer ≥10 mIU/mL), as well as children who were unprotected based on serology, but who had received a complete HBV vaccination according to their immunization cards.  3) To evaluate factors associated with having been vaccinated against HBV (regardless of serological status, protection or non-protection): an analysis was conducted in Cameroon and Senegal for children born in 2006 and after, i.e. at least one year after the integration of the HBV vaccine into the EPIs. CAR children were not included because the vaccine was integrated later. The variable "vaccinated" implied having received a complete HBV vaccination according to the immunization card, or if no immunization card existed, by anti-HBs+ and anti-HBc- status (with anti-HBs titer ≥10mIU/mL). All anti-HBs+ and anti-HBc+ children with no immunization card were removed because we could not know whether they were vaccinated but not protected, or if they were not vaccinated at all.  4) HBV current infection was estimated by the number of children with HBsAg-positivity |
| --- | --- | --- | --- | --- |
| Statistical methods | 12 | (*a*) Describe all statistical methods, including those used to control for confounding | Pages 6-7 | Continuous variables were expressed as medians and interquartile ranges (IQR) for and discrete variables as percentages.  For univariate and multivariate analysis, quantitative variables were expressed as dichotomous variables using either the median or a clinically relevant threshold.  Univariate analysis was based on the Fisher’s exact test for discrete variables and by analysis of variance or the Kruskal-Wallis test for continuous variables. All variables associated with “having been vaccinated” in univariate analysis (p<0.25) were included in a backward stepwise logistic regression model. A p value of ≤0.05 was considered statistically significant. Adequacy of the model was established through the Hosmer Lemeshow tests. Interactions between the variables found to be associated with “having been vaccinated” in the univariate analysis were tested using likelihood -ratio test. |
|  |  | (*b*) Describe any methods used to examine subgroups and interactions |  |  |
|  |  | (*c*) Explain how missing data were addressed |  |  |
|  |  | (*d*) *Cohort study*—If applicable, explain how loss to follow-up was addressed  *Case-control study*—If applicable, explain how matching of cases and controls was addressed  *Cross-sectional study*—If applicable, describe analytical methods taking account of sampling strategy |  |  |
|  |  | (*e*) Describe any sensitivity analyses |  |  |
| Results | | | | |
| Participants | 13* | (a) Report numbers of individuals at each stage of study—eg numbers potentially eligible, examined for eligibility, confirmed eligible, included in the study, completing follow-up, and analysed | Page 8 | 1783 consecutive children.  428 children with immunization card, 636 children documented for maternal records, 1696 documented for serology and 1739 documented for immunization card and serology |
|  |  | (b) Give reasons for non-participation at each stage |  |  |
|  |  | (c) Consider use of a flow diagram |  |  |
| Descriptive data | 14* | (a) Give characteristics of study participants (eg demographic, clinical, social) and information on exposures and potential confounders | Pages 8-10 | 1783 children were recruited: 763 in Cameroon, 535 in CAR and 485 in Senegal. No mothers of eligible children refused to participate in the study.Forty-four percent of the children were female, and the median age was 21 months [12-36 months]; children were significantly older in CAR.  The main causes of the children’s hospitalization were other infectious syndrome (33%) and gastro-intestinal infections (23%) in Cameroon; malaria in CAR (54%); and respiratory infections (28%) and other reasons (35%) in Senegal. |
|  |  | (b) Indicate number of participants with missing data for each variable of interest |  | NA |
|  |  | (c) *Cohort study*—Summarise follow-up time (eg, average and total amount) |  |  |
| Outcome data | 15* | *Cohort study*—Report numbers of outcome events or summary measures over time |  |  |
|  |  | *Case-control study—*Report numbers in each exposure category, or summary measures of exposure |  |  |
|  |  | *Cross-sectional study—*Report numbers of outcome events or summary measures | Pages 9-10 | Immunization coverage by country and by methods.  HBV infections by country |
| Main results | 16 | (*a*) Give unadjusted estimates and, if applicable, confounder-adjusted estimates and their precision (eg, 95% confidence interval). Make clear which confounders were adjusted for and why they were included | Page 10 | Overall HBV immunization coverage based on immunization cards was 99%, 49% and 100% in Cameroon, CAR and Senegal, respectively (p<0,001). The immunization rate based on maternal recall was 91%, 17% and 88% in Cameroon, CAR and Senegal, respectively (p<0,001). According to serology (anti-HBs titer≥10 mUI/mL and anti-HBc-), the coverage rate was 68%, 13% and 46% in Cameroon, CAR and Senegal, respectively (p<0,001). In Senegal and Cameroon, factors associated with having been vaccinated were: mother's higher education (OR=2.2; 95%CI[1.5-3.2]), no malnutrition (OR=1.6; 95%CI[1.1-2.2]), access to flushing toilets (OR=1.6; 95%CI[1.1-2.3]), and < 24 months old (OR=2.1; 95%CI[1.3-3.4] between 12 and 23 months and OR=2.7; 95%CI[1.6-4.4] < 12 months). The prevalence of HBV-infected children (HBsAg+) were 0.7%, 5.1%, and 0.2% in Cameroon, CAR and Senegal, respectively (p<0.001). 16% of anti-HBC children  Factors associated with having been vaccinated (regardless of protection). |
|  |  | (*b*) Report category boundaries when continuous variables were categorized |  |  |
|  |  | (*c*) If relevant, consider translating estimates of relative risk into absolute risk for a meaningful time period |  |  |

Continued on next page

| Other analyses | 17 | Report other analyses done—eg analyses of subgroups and interactions, and sensitivity analyses |  |  |
| --- | --- | --- | --- | --- |
| Discussion | | | | |
| Key results | 18 | Summarise key results with reference to study objectives |  |  |
| Limitations | 19 | Discuss limitations of the study, taking into account sources of potential bias or imprecision. Discuss both direction and magnitude of any potential bias | Page 13 | Selection of the population (hospitalized children with blood samples).  Cross sectional design |
| Interpretation | 20 | Give a cautious overall interpretation of results considering objectives, limitations, multiplicity of analyses, results from similar studies, and other relevant evidence | Page 13 | Comparison with data from WHO.  Comparison with other studies for factors associated with having been vaccinated (regardless of protection). |
| Generalisability | 21 | Discuss the generalisability (external validity) of the study results | Pages 13-14 | Variation of the immunization coverage according to the different methods : immunization card, maternal recall or serological profile.  Different estimations from WHO estimates when coverage was estimated with serological results  Low rate of HBs Carriers suggesting the effectiveness of the EPI |
| Other information | |  | | |
| Funding | 22 | Give the source of funding and the role of the funders for the present study and, if applicable, for the original study on which the present article is based |  | The study was funded by Pasteur Institute Network. |

*Give information separately for cases and controls in case-control studies and, if applicable, for exposed and unexposed groups in cohort and cross-sectional studies.

**Note:** An Explanation and Elaboration article discusses each checklist item and gives methodological background and published examples of transparent reporting. The STROBE checklist is best used in conjunction with this article (freely available on the Web sites of PLoS Medicine at http://www.plosmedicine.org/, Annals of Internal Medicine at http://www.annals.org/, and Epidemiology at http://www.epidem.com/). Information on the STROBE Initiative is available at www.strobe-statement.org.
